# The effect of policy measures, school holidays and travel on the incidence of SARS-CoV-2 infection in children and adults in Estonia from 2021 to 2022

**DOI:** 10.1371/journal.pone.0327719

**Published:** 2025-07-03

**Authors:** Hiie Soeorg, Taavi Päll, Aare Abroi, Radko Avi, Olga Sadikova, Mari-Anne Härma, Tuuli Reisberg, Irja Lutsar, Kristi Huik

**Affiliations:** 1 Department of Microbiology, Institute of Biomedicine and Translational Medicine, Faculty of Medicine, University of Tartu, Tartu, Estonia; 2 Institute of Technology, Faculty of Science and Technology, University of Tartu, Tartu, Estonia; 3 Department of Communicable Diseases, Health Board, Tallinn, Estonia; 4 Institute of Genomics, Faculty of Science and Technology, University of Tartu, Tartu, Estonia; Universitas Syiah Kuala, INDONESIA

## Abstract

**Purpose:**

The policy measures implemented during the COVID-19 pandemic, travel and school holidays could have influenced the spread of SARS-CoV-2 infection in children and adults differently. We aimed to determine the effect of policy measures, school holidays and travel on the incidence of SARS-CoV-2 infection in children and adults.

**Methods:**

The overall SARS-CoV-2 infection incidence between 1 February 2021 and 1 May 2022 was decomposed into the most common lineage-specific incidence per 100,000 by imputing lineage based on the sequencing results of random samples. A phenomenological logistic growth model was fitted to the incidence data in adults (aged ≥15 years) and children (aged <15 years) in four regions in Estonia. Factors influencing the spread of the virus, such as policy measures, school holidays, the number of travel-related cases, and cumulative vaccination or infection rates, were tested as covariates in the model.

**Results:**

The eleven most common lineages (one Alpha, six Delta, two Omicron BA.1, and two Omicron BA.2) caused 84.7% and 85.8% of all SARS-CoV-2 infections in children and adults, respectively, during the study period. According to the final model, the Delta variant had slower growth and a lower maximum cumulative incidence. The number of workplace closures in the previous week and school holidays in the same week decreased, but the number of travel-related cases in the same week increased the incidence growth. No difference between children and adults was observed. The testing rate was lower during school holidays than during school terms (median (IQR) 1964 (1437–2970) vs. 3136 (2476–4417) vs. per 100,000; p < 0.001). In 40.3% of the weeks, travel-related cases were detected, accounting for (IQR) 2.1% (0.9–4.3%) of the incidence of nontravel-related infections.

**Conclusion:**

Our study suggests that the spread of SARS-CoV-2 infection was similar in children and adults. Workplace closures reduced transmission, whereas during school holidays lower testing contributed to a lower reported incidence, and travel-related cases were possibly underreported.

## Introduction

The policy measures implemented during the COVID-19 pandemic, such as workplace or school closings and different vaccine rollouts for different age groups, could have influenced the spread differently in children and adults. Additionally, school holidays have been proposed to have reduced the transmission due to fewer contacts [1,2]. In contrast, school holidays increased the incidence in the 12–17-year age group, possibly due to travel [2], warranting the consideration of mobility in determining its impact. Understanding possible differences in the impact of the pandemic on various demographic groups could help in designing targeted interventions to mitigate the effects of a pandemic more effectively [3].

The effect of international travel on spread has been controversial, with some suggesting its role in increasing incidence [4], whereas others emphasize its importance only in the introduction of the virus but not in the local spread [5,6]. Such differences could be due to the combination of different lineages and mobility from various countries. The importation of the virus leads to a wave, which, in the case of the introduction of different lineages, is caused by a variety of lineages [7]. The resulting incidence curve is a composite of the spread of multiple lineages warranting stratification of the data [8]. Failure to decompose aggregate incidence curves into specific incidences leads to difficulties in capturing the effect of other factors on transmission [9]. Correspondingly, during the concomitant spread of multiple lineages, mobility in relation to the specific lineage should be considered; in such situations, the importance of overall mobility diminishes compared with the extensive spread of a dominant variant [10].

This study aimed to assess the impact of policy measures, school holidays, and travel on the incidence of SARS-CoV-2 infection in children and adults by disaggregating overall incidence into lineage-specific trends and distinguishing travel-related importation of specific variants.

## Materials and methods

### Study data

This study is based on two data sources. First, the reported incidence of SARS-CoV-2 infections by the Estonian Health Board [11] was used, from which the number of SARS-CoV-2 infections by county and age groups for the period between 1 February 2021 and 1 May 2022 was obtained. Second, a nationwide SARS-CoV-2 whole-genome sequencing dataset in Estonia [12] was accessed, from which lineages from sequences of randomly selected samples of nontravel-related cases and all lineages of travel-related cases in the same period were obtained. Travel-related cases were defined as SARS-CoV-2 PCR-positive tests from people who had been in a foreign country within the prior two weeks. During the periods between 1 and 6 February 2021, 27% and between 6 and 15 January 2022, 41% of all travel-related cases were sequenced. For subsequent analysis, non-sequenced samples were assumed to contain each lineage with the same proportion as sequenced samples.

For the analysis, the two datasets were divided by an age cut-off of 15 years (<15 years hereafter referred to as children and ≥15 years hereafter as adults, to align with population statistics on population size) and region – the three largest counties (Harju, Tartu, Ida-Viru) in terms of population size and the remaining counties (Other). Thus, the spread of each lineage is included in the final dataset eight times (for each age group–region combination).

The analysis uses only the most common lineages, which comprised >5% of all sequences in at least one week in all four regions, similar to our previous analysis of the dynamics of the proportions of these lineages among sequences [13]. Here, the overall incidence was decomposed into lineage-specific incidences by allocating reported cases to the lineages proportional to the distribution of the lineages in the sequencing data in the same week, age group, and region. If there were ≤10 sequences available, the lineages were designated according to the multinomial logistic regression model developed in our previous work [13].

The daily incidences were aggregated to weekly incidences per 100,000. The number of travel-related cases was subtracted from the reported incidence to allow use of the number of travel-related cases as a proxy for mobility. The incidence data were subsequently transformed to a 5-week moving average to smooth the data for stable estimation of the parameter values [14]. As the first cases of a lineage can be followed by many days without detection before rapid spread in the community, the model can have a poor fit to the data for such stochastic events [15]. Thus, the incidence data for modelling each lineage in each region and age group started when a maximum of 2 weeks was observed without detecting the same lineage. To exclude second waves due to local outbreaks, the weeks following more than a 1.5-fold increase in the incidence after the peak were removed from the data. Only lineages with >5 observations and a maximum incidence >10 per 100,000 were included to improve the stability of the model.

### Statistical analysis

#### Model.

For the description of the incidence, a model based on a logistic curve to cumulative incidence was used [16]: $I(t)=\frac{dC(t)}{dt}=I_{0}{C(t)}^{\alpha }{\left(1-\frac{{C(t)}^{\gamma }}{K}\right)}^{\beta }$, where I(t) is the incidence in week t, C(t) is the cumulative incidence by week t, $I_{0}$ cases are observed when spread is detected, α is the growth parameter, K is the maximum cumulative incidence, the parameter β is the rate of approach to the plateau and γ is the asymmetry parameter [16]. From this equation, a differential equation was obtained, $\frac{dI(t)}{dt}=\left(\frac{\alpha }{C(t)}-\frac{\beta^{\gamma }}{K^{\gamma }-{C(t)}^{\gamma }}\right){I(t)}^{2}$, which was used for fitting the model to the data. Between-lineage, between-age group and between-region variability was included in the model as log-normally distributed random effects for α and K: $\alpha =\theta_{\alpha }e^{\eta_{i,j}+\eta_{k}}$, $K=\theta_{K}e^{\xi_{i,j}+\xi_{k}}$, $\eta_{i,j}\sim N\left(0,\sigma_{1}\right)$, $\eta_{k}\sim N\left(0,\sigma_{2}\right)$, $\xi_{i,j}\sim N\left(0,\psi_{1}\right)$ and $\xi_{k}\sim N\left(0,\psi_{2}\right)$, where $\theta_{\alpha }$ and $\theta_{K}$ are the population average values. The initial value of the incidence was set as the first reported weekly incidence, $I(0)=I_{0}$, to which log-normal variability was included for the initial value of the cumulative incidence: $C(0)=I_{0}e^{\mu_{i,j,k}}$, $\mu_{i,j,k}\sim N(0,\phi )$. A proportional residual error model with errors from normal distribution was used.

The following variables were tested as covariates of the growth parameter α: age group (children, adults); variant (Delta, Omicron); policy measures (school closing, workplace closing, cancelling public events, restrictions on gatherings, stay-at-home requirements, restriction on internal movement between regions, policy on contact tracing, policy on facial coverings, testing policy) from the Oxford Covid-19 Government Response Tracker [17]; school holiday; number of travel-related cases per 100,000; cumulative vaccination rate and cumulative infection rate from the Estonian Health Board [11] and their 1- or 2-week lagged values; population size and density from Statistics Estonia [18]; rate of change in the number of SARS-CoV-2 tests performed or the proportion of positive tests [11]; the prevalence of other lineages at the same time; and seasonality as a sine function with a maximum in winter and a minimum in summer [19]. For the maximum cumulative incidence K, age group, variant, cumulative vaccination and infection rate at the beginning of the spread, population size and density and the number of detected cases per 100,000 (travel- or nontravel-related cases) before the spread in the community were tested as covariates. For β and γ, age group and variant were considered covariates. Covariates were included in the model if they reduced the −2 log-likelihood by at least 3.84 (corresponding to p < 0.05), resulting in coefficients statistically significantly different from 0 (if the parameter in the exponent, e.g., θ in the case of $\alpha ,K={covariate}^{\theta }$) or 1 (if the parameter linearly related to growth or maximum cumulative incidence, e.g., θ in the case of $\alpha ,K=\theta^{covariate}$), and did not result in zero gradients in estimation.

#### Simulations of cumulative incidence.

To describe the effect of covariates influencing the growth parameter, the scenarios for increasing restrictions or reducing the number of travel-related cases for 4 or 8 weeks from the week when the incidence increased over 25, 50 or 100 per 100,000 were considered. The cumulative incidence during the time of observed spread was calculated for each scenario.

For the model fitting and simulations, NONMEM (version 7.5; ICON Development Solutions, MD, USA) was used. All other analyses were performed in R (version 4.3.2; R Foundation for Statistical Computing, Vienna, Austria) using packages readr, readxl, dplyr, reshape2, ggplot2, ggpubr, paletteer, showtext, xpose, xpose4, nnet, and splines.

The analysis was approved by the Research Ethics Committee of the University of Tartu (approvals 304/T-1, 324/T-1, 376/M-7). Informed consent was not required according to § 6 of the national ‘Personal Data Protection Act’.

## Results

**Fig 1:**
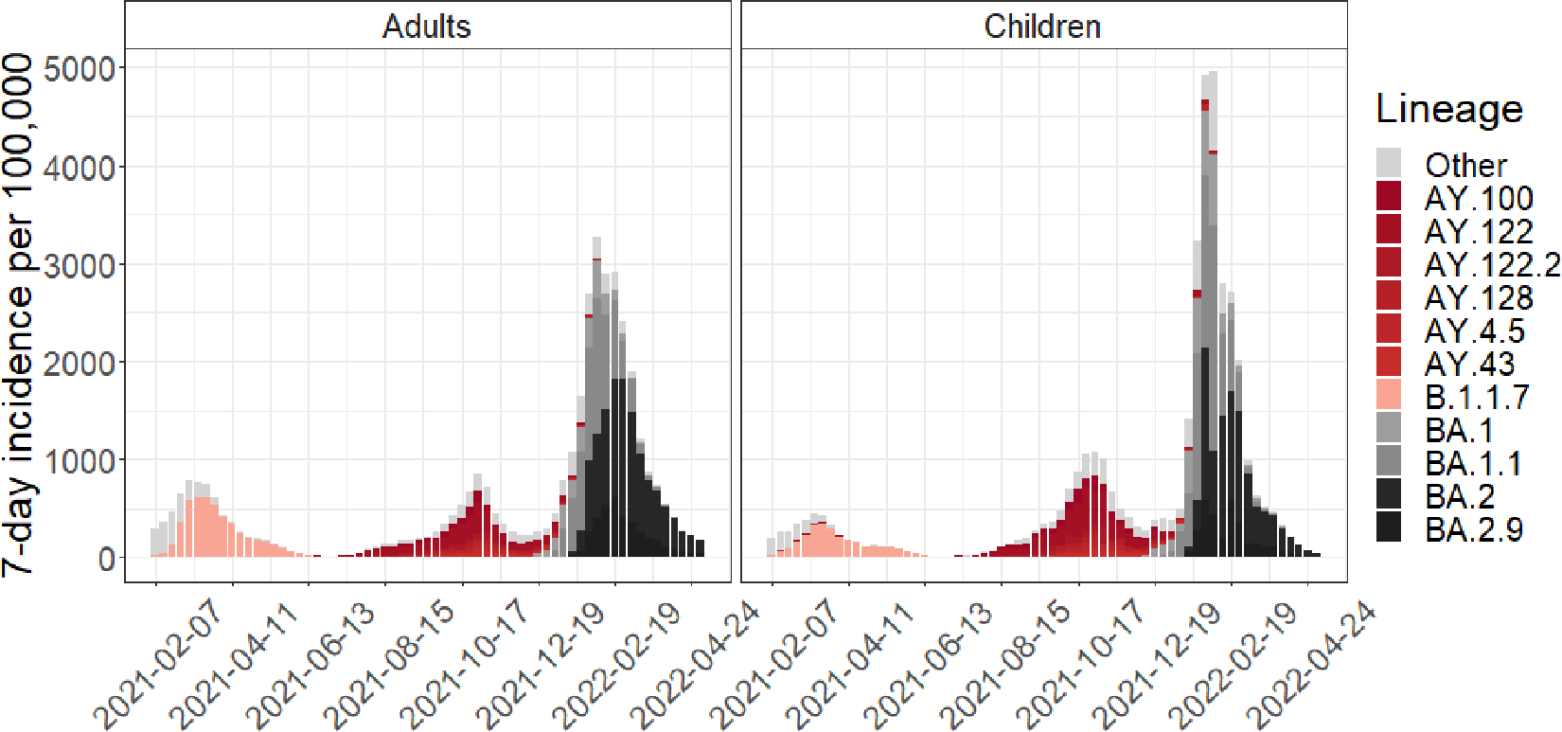
Weekly incidence per 100,000 of SARS-CoV-2 infections in adults (≥15 years) and children (<15 years). The incidence is decomposed into lineage-specific incidences of the eleven most common lineages and other lineages.

During the study period, three waves (Fig 1) occurred during which 513,609 cases were reported: 87,615 in 218,102 children and 425,994 in 1,111,009 adults. The incidence during the Delta and Omicron spread was composed of superimposed waves of distinct lineages, of which 11 were designated as the most common and accounted for 85.8% of the cases in adults and 84.7% in children. The median (interquartile range, IQR) maximum cumulative incidence in the regions and age groups ranged from 712 (478–1301) per 100,000 in the case of Delta to 6162 (2236–9452) in the Omicron BA.2 lineages, and the maximum incidences of 118 (76–206) and 1145 (438–1623) per 100,000, respectively, and were not statistically significantly different between children and adults.

Overall, school holidays comprised a median (IQR) of 25% (18–40%) of the time of lineage was spreading. The recommended or required closing of workplaces was in place at a median (IQR) of 71% (48–95%) of the time. School closures were required only during Alpha lineage spread, and restrictions on gatherings were in place during the Alpha lineage and at the beginning of the Delta AY.122 lineage. The median cumulative vaccination rate ranged from 0% to 8.9% in children and from 1.6% to 72.2% in adults, and the cumulative infection rate ranged from 1.4% to 17.5% and 3.5% to 20.3%, respectively. The median (IQR) weekly testing rate was 2889 (1820–4042) per 100,000 and the proportion of positive tests was 14.1% (9.0–24.8%). The median (IQR) ratio of the testing rate over two consecutive weeks was 1.00 (0.87–1.12). Fewer tests were performed during school holidays than during school terms (median (IQR) 1964 (1437–2970) vs. 3136 (2476–4417) per 100,000; p < 0.001) and during required workplace closings compared with no or only recommended closings (median (IQR) 1922 (1363–2567) vs. 3026 (1964–4199) per 100,000; p < 0.001), similarly in children and adults. In 40.3% of the weeks included, travel-related cases were detected, with a median (IQR) incidence of 2.1% (0.9–4.3%) of the nontravel-related cases, which was similar in children and adults.

**Fig 2:**
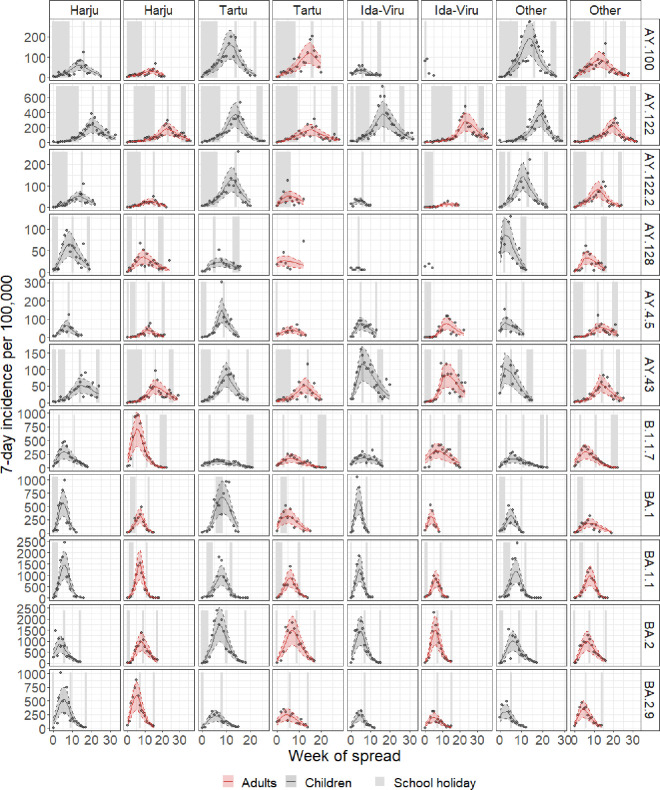
Individual predictions from the final model. The observed incidences of the most common lineages in the four regions and adults (red) and children (grey) and model-predicted incidences over the time of spread are shown. Solid lines represent individual predictions, and the shaded area around them bounded by dashed lines represents the 95% prediction interval. The grey shaded areas represent the timing of school holidays.

In the final model, the parameter for the rate of approach to the plateau β and the asymmetry parameter γ were fixed to 1, and the growth parameter and maximum cumulative incidence were smaller in the case of Delta than in the cases of Omicron and Alpha (Table 1). The growth parameter, adjusted for the change in the testing rate, was smaller in the case of a school holiday occurring in the same week and a workplace closing in the previous week. The number of travel-related infections in the same week increased the growth parameter, with the influence being smaller in the case of Omicron BA.2 than in the other lineages. There was no difference in the parameter values or differential effect of the covariates between adults and children. The model had a good fit to the data (Fig 2).

**Table 1:** Parameters of the final model.

Parameters with covariates	Parameter	Estimate (relative standard error)
$\alpha =\theta_{\alpha }\cdot$	$\theta_{\alpha }$	1.61 (14%)
$(1+work{)}^{\theta_{work}}\cdot$	$\theta_{work}$	-0.90 (19%)
${\theta_{school}}^{schoolholiday}\cdot$	$\theta_{school}$	0.68 (7%)
${\left(1+\frac{N_{travel}}{I(t)}\right)}^{\left(\theta_{travel}\cdot (1-BA\mathrm{.2})+\theta_{BA\mathrm{.2}}\cdot BA\mathrm{.2}\right)}\cdot$	$\theta_{travel}$	13.66 (21%)
	$\theta_{BA\mathrm{.2}}$	1.86 (33%)
${\left(\Delta N_{t}\right)}^{\theta_{Ntests}}\cdot$	$\theta_{Ntests}$	-0.23 (42%)
${\theta_{\alpha \_Delta}}^{Delta}\cdot$	$\theta_{\alpha \_Delta}$	0.62 (15%)
$e^{\eta_{i,j}+\eta_{k}}$	$\sigma_{1}$ $\sigma_{2}$	0.13 (15%) 0.12 (14%)
$K=\theta_{K}\cdot$	$\theta_{K}$	4330 (9%)
${\theta_{K\_Delta}}^{Delta}\cdot$	$\theta_{K\_Delta}$	0.22 (13%)
$e^{\xi_{i,j}+\xi_{k}}$	$\psi_{1}$ $\psi_{2}$	0.37 (8%) 0.59 (7%)
$C(0)=I_{0}\cdot e^{\xi_{i,j,k}}$	ϕ	0.81 (15%)
Residual proportional error	Standard deviation	0.24 (4%)

C(0) – the initial value of the cumulative incidence; BA.2 – Omicron BA.2 (0 = no, 1 = yes); Delta – Delta (0 = no, 1 = yes); $I_{0}$ – the initial value of the incidence (the observed incidence in the first week of spread); $\Delta N_{t}$ – number of tests performed in the same week/number of tests performed one week before; $N_{travel}$ – number of travel-related cases per 100,000 in the same week; schoolholiday – school holiday during the same week (0 = no, 1 = yes); work – workplace closure one week before (0 – no measures, 1 – recommended closing, 2 – required closing for some sectors, 3 – required closing for all-but-essential workplaces) Subscript i indicates the age group, j region, k lineage.

**Fig 3:**
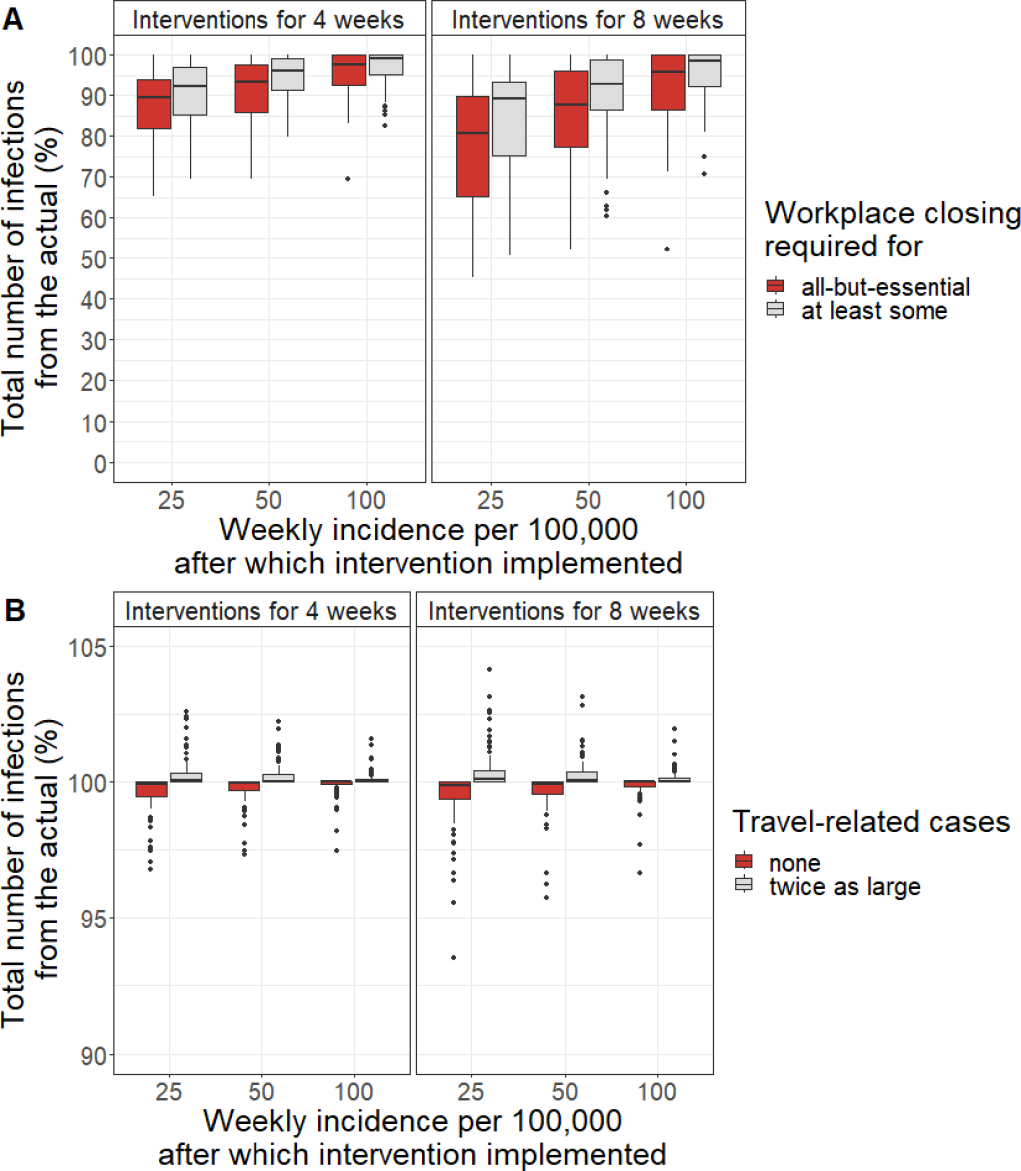
Simulations of cumulative incidence rates. Simulated total number of infections compared with the actual total number of infections (A) if workplace closure had been at least at the level required for some or all-but-essential sectors and (B) if no travel-related cases or twice as many travel-related cases had occurred.

According to the simulations, if workplace closure had been required for at least some sectors or required for all-but-essential sectors, the total number of infections during the same time would have decreased by a median of 10.9% and 19.3%, respectively, if it had been implemented for 8 weeks after the incidence increased above 25 per 100,000. Later implementation and/or implementation for only 4 weeks would have decreased the number of infections up to 7.7% and 12.5%, respectively (Fig 3A). If no travel-related infection had occurred or if the number of travel-related cases had been twice as large, the median total number of cases would have remained the same (Fig 3B).

## Discussion

In this study, we showed that the spread of the most common Alpha, Delta and Omicron BA.1 and BA.2 lineages was similar among children and adults. The lagged effect of workplace closure suggests its effectiveness in reducing the spread, whereas the impact of school holidays and the number of travel-related cases on the incidence in the same week could be attributable to reduced testing or underreporting of the infection, respectively.

The effectiveness of workplace closure on the growth of the incidence is expected, as it reduces mobility [20] and thus contacts within the population [21] and potential transmission [6]. Furthermore, earlier implementation of a measure is more effective in limiting the spread [7,22], as our study confirmed. However, the effect of the intervention has been shown to take 2–2.5 weeks [21,23]. An estimated lag of 35 days has been reported for the effect of workplace closures during 2020 in European countries, including in a group containing Estonia [24]. In contrast, even the short-term closing of companies has been effective [25]. In our study, the estimate for the effect of workplace closure is affected by stricter restrictions during spring 2021. Therefore, more than half of the people were in favour of stricter measures [26] and thus possibly more people readily contributed to reducing the spread. This is in line with the context-dependency of the effectiveness of interventions [24].

Various measures concerning the effectiveness of workplace closures have been used by others, such as reduction in the reproduction number [27,28], a reduction in the number of cases [29], and a model parameter showing effectiveness [30]. Different measures make comparison of the effectiveness between studies difficult. Nevertheless, with respect to relative effectiveness, others have shown that school closures [27–30] limit spread more effectively than workplace closures. In our dataset, the school closure was in place only for a short time, which probably hindered the identification of this measure as a significant factor. Similarly, the number of weeks when restrictions on gatherings were in place was small in our study and could have contributed to this measure not being found effective in our study. While ineffectiveness of such restrictions is in line with findings in some studies [29,30], it contradicts another study in which restrictions on gatherings were more effective than workplace closure [27]. Although we found that workplace closures were effective in reducing the spread, the number of tests performed during these periods was reduced. Thus, our study findings add an additional aspect of the impact of testing capacity on the effectiveness assessment – it may co-vary with the restrictions.

The better fit of the non-lagged effect of school holidays to the growth suggests its impact on the incidence through reduced testing rather than by reducing the spread. Indeed, as school holidays affect only schools and the closing of other places is independent of school holidays, the effect of school holidays on the spread is less pronounced than that when other places are closed as well [21]. We found a better fit of the non-lagged effect of school holidays to growth and a decreased number of tests performed during school holidays. These findings suggest a reason for the reduced incidence during school holidays – reduced testing which can also explain findings from other authors. A reduced spread in children younger than 12 years of age during school holidays was observed in a recent study [2]. However, the authors also discussed the possible impact of not considering variability in the testing rate [2]. In another recent study, the authors assumed reduced contact during school holidays [31]. However, their results were not robust to changing contact rates during school holidays, leaving uncertainty around how school holidays impact incidence. To date, the increasing effect of school holidays on incidence has been suggested to be due to travel [2,32,33]. The reduced number of tests performed during school holidays in our study may be partly in line with this hypothesis. The hypothesis is supported by there being no differential effect of school holidays on the spread or testing rates in children and adults, suggesting common behavioural changes with reduced testing.

The role of differences in age groups in the spread of the virus is still under investigation [34]. Our study suggests that no differential measures would be needed for children and adults. Our analysis during the pandemic from Alpha until Omicron BA.2, a period in which schools were open for most of the time, revealed that the spread was similar in children and adults. Owing to lower contact rates between children and adults than between children [35], the significant contribution of children to transmission could manifest as faster or greater spread. However, we did not see that. This finding shows that although the contribution of children may be important after schools are reopened [34], special measures to reduce the spread among children are not warranted when schools are already open. Notably, we also did not detect a differential impact of workplace closures on the incidence. Thus, transmission among children is efficiently reduced by workplace closures. Similarly, remote working was also found to be protective for children [1], which could be due to reduced mobility [20] and thus reduced contact [21].

The number of travel-related cases increased the growth parameter during the same week. This may suggest underreporting of travel-related cases, i.e., visiting a foreign country was not recorded for all confirmed cases, rather than the effect of travel on the transmission. The effect of travel on the spread of a virus is usually described by modelling incidence as a function of a measure of mobility [6,36,37] or by phylogenetic analysis [38,39]. Such studies have shown no effect of import via international travel on the incidence growth rate [36] and no effect from the fact that up to half of the imported strains do not descend in the destination country [39]. The lag between the estimated import intensity, which combines the travel and strain distributions at the origin, can reach 12 weeks [37]. Increased international travel increases diversity [36]. These findings suggest that the importance of importation is in its role in the establishment of the virus which subsequently leads to local spread [6]. However, varying timing of the spread of specific lineages between countries [13,40] renders the number of passengers noisy. Not all cases are sequenced to determine the spread of imported strains in the country by phylogenetic analysis. Thus, the linking of various data sources is needed. We approached this by combining phylogenetic information, the number of imported cases and the incidence data. Our results of no lagged effect of travel restrictions confirm the speculations arising from phylogenetic studies: the benefits of travel restrictions are limited to reducing the risk of importation, and if implemented later, their effectiveness is limited [38,39].

The differential effect of Omicron BA.2 on the number of travel-related cases was included due to previous findings; that is, unlike other variants, Omicron BA.2 was not detected among travel-related cases earlier than among nontravel-related cases, suggesting infection in Estonia, possibly due to its rapid spread locally [13]. Similarly, the effect of the number of travel-related cases in the same week could be partially due to these cases merely reflecting spread in Estonia. As we defined travel-related cases as infections from those who had visited a foreign country within the last 14 days, and this period is longer than the estimated lag time of 8 days between importation and the first detected local transmission event [7], we cannot exclude some impact of travel on transmission. However, even if possible effects on transmission are assumed, our simulations showed a minimal effect on transmission. Nevertheless, the estimated effect of travel must be considered in the context of restrictions applied to travellers. The proportion of imports from abroad leading to transmission decreases after the implementation of quarantine [6]. During the periods of required negative tests before travelling, the correlation between mobility and incidence decreases [41].

Contrary to studies on the effectiveness of previous infection (65%) [42] or vaccination (77%) [43] in protecting against subsequent infection [2,22,36], we did not find such an impact. The reason could be the presence of a large reservoir for persistent transmission formed by uninfected and unvaccinated populations [36]. The importance of people without immunity in the incidence data is confirmed by national data, which show that two-thirds of cases at the time of the start of Omicron spread were individuals who were not vaccinated or had not had previous infection [44]. Second, immune evasion and declining immunity reduce the impact of previous infection and vaccination on spread [36,40]. The protection by vaccination or previous infection against infection is incomplete, particularly against asymptomatic infection compared with symptomatic infection (60% vs. 85% in cases of previous infection [42] and 45% vs. 77% in cases of vaccination [43]). Furthermore, the effectiveness of vaccination wanes from 80% to 53% for pre-Omicron strains and from 38% to 17% for Omicron strains within the first 3 and at 6 months after the vaccination, respectively [45]. Different levels of protection from previous infection also vary over time – 89% in the case of Alpha, 81% in the case of Delta and 39% in the case of Omicron [42]. The need to develop new vaccines against emerging variants also highlights suboptimal protection by immunity [46].

We also did not find an effect of seasonality, i.e., a lower spread in summer and higher spread in winter [1,9]. This is contrary to a time series analysis based on data from the United States and Europe from March 2020 to December 2022, including Estonia, which reported seasonal peaks in COVID-19 between November and April [47]. We may have missed this in our analysis because the Delta variant had a lower growth rate and cumulative incidence parameter value, but it also spread in summer and autumn when transmission could be lower. Thus, the lower parameter values may be due to its overall lower transmissibility [15,48] and lower spread in summer. To separate the effect by variant and season, we tested seasonality as a sine function with fixed values based on German data [19]. Although Estonia is a country with a temperate climate, we cannot exclude the possibility that slightly lower temperatures in Estonia would require somewhat different parameter values. Our analysis included only one summer and one winter, which may not be sufficient to detect seasonality, particularly in the case of correlations between variants and seasons.

Some limitations of our study must be noted. First, the predicted number of lineage-specific infections was based on a small number of sequences and a small number of cases if stratified by age group and region, leading to uncertainty and imprecision in the data. However, describing the same lineage in multiple regions and two age groups with random effects in the model accounts for such variability in data subsets and minimizes potential biases compared with that when only one dataset is used [15]. Second, due to the lack of such data, we did not consider mobility between regions within Estonia, which is an important driver of infection transmission [21,22]. However, this is accounted for by the nested random-effects structure, where each lineage has a common random effect for all regions, while the random effect for each county still accounts for variation in the spread between regions due to sociodemographic factors, for example [7,15,49]. As the importation of lineages rather than mobility itself is important for fuelling transmission, exemplified by the offset of less mobility by greater incidence [22], this model structure partly accommodates the effect of mobility as well. Third, unlike SIR-type models, the phenomenological model did not allow us to delineate the effect of interventions on specific aspects of infection spread, such as transmission and susceptibility. However, in the case of scarcity and uncertainty in the data, SIR-type models may not work well [50]. Furthermore, by using the testing rate change covariate, we adjusted the incidence for changes in testing, for which the importance on the incidence has been noted [2,8].

In conclusion, the spread of SARS-CoV-2 lineages was similar in children and adults. The study suggests that workplace closure reduced transmission, but the apparent reduced spread during school holidays was attributable to lower levels of testing. Travel, once a lineage spreads in the country, had minimal effect on the incidence. Our findings suggest the following recommendations for future public health strategies which are similar for children and adults. First, workplace closures reduce transmission, whereas earlier implementation is more effective. Second, the reduced incidence during school holidays is apparent and due to reduced testing. Third, travel has little effect on overall incidence in the community once the spread has been established.
